# The Relation Between Social Anxiety and Perceptions of Likeability and Friendship in Adolescents

**DOI:** 10.32872/cpe.10705

**Published:** 2024-12-20

**Authors:** Jeanine M. D. Baartmans, Bonny F. J. A. van Steensel, J. Loes Pouwels, Tessa A. M. Lansu, Reinout W. H. J. Wiers, Susan M. Bögels, Anke M. Klein

**Affiliations:** 1 UvA Minds: Academic Treatment Centre, Amsterdam, The Netherlands; 2 Developmental Psychology, University of Amsterdam, Amsterdam, The Netherlands; 3 Education and Child Studies, Leiden University, Leiden, The Netherlands; 4 Child Development and Education, University of Amsterdam, Amsterdam, The Netherlands; 5 Behavioural Science Institute, Radboud University Nijmegen, Nijmegen, The Netherlands; 6 Developmental and Educational Psychology, Leiden University, Leiden, The Netherlands; Friedrich-Alexander-Universität Erlangen-Nürnberg, Erlangen, Germany

**Keywords:** social anxiety, adolescents, worry, avoidance, likeability, cognitive bias, friendship

## Abstract

**Background:**

This study investigated how different social anxiety symptoms (i.e., worrying about negative evaluation versus avoidance tendencies) in adolescents are related to the perception accuracy of likeability by peers and friendships with peers.

**Method:**

A community sample of 263 adolescents between 12 and 15 years old reported on their social anxiety symptoms. In addition, they estimated how much their peers liked them, indicated how much they liked their peers, and who their friends were in their classroom.

**Results:**

Results showed that socially anxious adolescents who mainly worried about negative evaluations, underestimated their likeability by peers. Adolescents with strong social avoidance tendencies had a more accurate perception of their likeability and friendships; they were less liked by their peers and had fewer friends.

**Conclusion:**

The results emphasize the importance of treating avoidance behavior in social anxiety since avoidance tendencies may not only maintain the social anxiety symptoms but are also related to a more negative judgment by others.

## Highlights

- Adolescents who worry about their social evaluation tend to underestimate their own likeability.
- Adolescents who tend to avoid social situations are less liked and have fewer friends.
- It is important to be aware of different symptoms of social anxiety.

## Background

The beginning of adolescence is characterized by an increased sensitivity to others’ judgment. Adolescents find it important to be liked by their peers and to be accepted in the peer group and often fear being rejected or negatively evaluated. Therefore, fear of negative evaluation is part of normal development. Still, it can become problematic when these fears hinder adolescents in their daily lives and development (American Psychiatric Association, 2013). Several studies indeed found that a strong fear of negative evaluation peaks in early adolescence due to challenges in the peer context and social-cognitive development. This heightened fear of negative evaluation, one of the key cognitive symptoms of social anxiety, can amplify peer relationship stress (Chavira & Stein, 2005; Erath et al., 2008; Ranta et al., 2014).

However, fear of negative evaluation is only one of the symptoms associated with social anxiety, with other symptoms including attentional, emotional, behavioral, and physical symptoms also being present in social anxiety. In this study we focused on cognitive symptoms (fear of negative evaluation) and behavioral symptoms (avoidance). With regards to cognitive symptoms, adolescents with social anxiety (disorder) have strong assumptions about being judged or rejected by others (American Psychiatric Association, 2013; Bögels et al., 2010); they have fears of saying something 'wrong,' behaving 'inappropriate' or making a fool out of oneself in social situations. In addition, they expect that their social performances have disastrous consequences, and as a result, they are likely to avoid social situations (Leigh & Clark, 2018; Ranta et al., 2014). Thus, cognitive symptoms often lead to behavioral symptoms: i.e., avoidance of feared (social) situations and/or the use of safety behaviors to avoid the perceived anxious scenario from happening (e.g., extremely preparing and/or memorizing what to say prior to giving a presentation because the child believes this will prevent that other children will make fun of him). The avoidance of threatening stimuli results in a decrease in anxiety in the short term. However, in the long term, avoidance may maintain social anxiety by preventing habituation and by disconfirmation of biased perceptions (Miers et al., 2014; Rapee & Spence, 2004). For social anxiety specifically, social avoidance may be reflected in withdrawal from peer interactions. The current study aims to examine the associations of (the subtypes of symptoms of) social anxiety with social functioning and (biased) perceptions of social functioning.

There is a growing body of evidence showing that adults with social anxiety (disorder) also suffer from actual deficits in social interactions (e.g., Voncken & Bögels, 2008). In addition, social withdrawal has been associated with social anxiety in children and can be defined as abstaining from social activities in the presence of peers (Erath et al., 2007). This withdrawn behavior prevents adolescents from practicing their social skills (Blöte et al., 2014; Clark & Wells, 1995). However, instead of, or in addition to actual lesser social performance, individuals high in social anxiety might also underestimate their social performance. Cognitive theories state that people with high levels of social anxiety or social anxiety disorder have a negatively biased perception of their social performance (e.g., Clark & Wells, 1995; Hofmann & DiBartolo, 2014; Rapee & Heimberg, 1997). As a result, it could be that the relation between social anxiety and worse social performance in self-report measures is affected by negative cognitive biases and might not be in line with others’ opinions. Regarding youths, research shows considerable evidence for the relation between social anxiety, negative expectations of one’s own social performance, and negative ratings of one’s own social competence (Kingery et al., 2010).

In previous studies, we tested the association between social anxiety and the accuracy of being disliked by peers in children and adolescents (Baartmans et al., 2019, 2020; Klein et al., 2018). For example, Baartmans and colleagues (2019) examined the extent to which children between 7 and 13 years old had an accurate or biased perception of their general likeability among classroom peers. Results showed that in children, higher levels of social anxiety were associated with underestimating one’s likeability among classroom peers. In line with this study, Baartmans and colleagues (2020) examined these questions in a different sample, using the same age group (7 to 13 years old) but investigated estimations of likeability among each peer individually instead of likeability among all classroom peers. Results again showed that when children had higher levels of social anxiety, they more strongly underestimated their own likeability. In addition, it was found that older children with social anxiety symptoms were more likely to underestimate their likeability by peers than younger children (Baartmans et al., 2020). Finally, Klein and colleagues (2018) followed a similar procedure to study adolescents aged between 12 and 19 with a mild intellectual disability. They also found that social anxiety symptoms were linked to a biased perception of likeability; adolescents with mild intellectual disability and with symptoms of social anxiety underestimated their likeability by peers (Klein et al., 2018). Previous research thus showed preliminary evidence for a relation between social anxiety and a negatively biased perception of likeability. In order to be able to compare the findings of the current study to previous findings, the current study examines a group of typically developing adolescents with an age range in between the studies mentioned above (10-15 years).

Whereas we already have some insight into how social anxiety is related to youth’s (accuracy of the perception of) likeability among peers, we know less about their functioning and particularly accuracy regarding friendships. In addition to likeability, friendship plays a vital role in the lives of adolescents. Although both constructs are closely related (Cantin et al., 2019), they each tap into different components of social competence. Whereas likeability reflects a relatively general affective evaluation, friendships, conversely, are dyadic relationships requiring mutuality (Greco & Morris, 2005). Research on the relation between social anxiety and friendships in youth showed that social anxiety negatively influences companionship and intimacy in friendships (Vernberg et al., 1992) and that higher social anxiety is related to negative interactions in best friendships (La Greca & Harrison, 2005). Studies that focused on the number of friends about social anxiety symptoms suggested that especially girls with high levels of social anxiety report fewer friendships and less intimacy and support within close friendships (La Greca & Lopez, 1998). Socially anxious adolescents also indicate to have fewer friends, and they more often choose other socially anxious youth as their friends (Van Zalk et al., 2011; but see Karkavandi et al., 2022, who find that socially anxious girls nominate as many friends as socially non-anxious girls).

However, important to note is that most findings are based on self-report measures. This raises the question of to what extent these findings reflect actual friendship functioning or are driven by negative cognitive biases related to social anxiety. There are some indications that socially anxious youth may indeed be less likely to be selected as a friend (Karkavandi et al., 2022; Van Zalk et al., 2011) and that they have fewer reciprocated friendships (Erath et al., 2010). However, it could also be the case that adolescents do not always recognize it when peers consider them to be friends (an underestimation of friendship). Therefore, the second goal of this study was to investigate the association between social anxiety symptoms in adolescents and the accuracy of their estimations of friendships within the classroom.

## The Present Study

The overall aim of this study was to examine the relations of social anxiety level with cognitive biases and social performance in the peer group among a large group of adolescents (10-15 years old). We examined this aim in the context of likeability and friendships within a classroom setting. In addition, we paid special attention to the subcategories of social anxiety symptoms; i.e., cognitive symptoms (fear of negative evaluation) and behavioral symptoms (avoidance).

To answer the first research question – how social anxiety symptoms in adolescents are related to self-perceived and peer-perceived likeability and the perception accuracy of likeability by peers - we first studied how both self-perceived and peer-perceived likeability were related to social anxiety in adolescents (Goal 1a). We expected that social anxiety would be negatively related to self- and peer-perceived likeability. Regarding the subcategories of social anxiety, we expected that especially the behavioral symptoms (avoidance) would play an important role in actual likeability among peers because avoidance prevents adolescents from practicing their social skills, and adolescents with social skills deficits can be less likeable (Blöte et al., 2012, 2014; Clark & Wells, 1995; Miers et al., 2010, 2011). Moreover, when socially anxious adolescents avoid social interactions with peers, these peers are less likely to get to know them. They may, therefore, be less likely to develop a positive opinion about them. This is also in line with the findings of Henricks and colleagues (2021, 2023), who find a negative association between the behavioral social anxiety component ‘avoidance’ and likeability among peers, but not between the cognitive ‘fear of negative evaluation’ component and likeability. In Goal 1b, we focused on the discrepancy between the two reporters and its relation with social anxiety symptoms in adolescents. Based on previous research in children, we hypothesized that higher levels of (cognitive symptoms of) social anxiety in adolescents would be associated with stronger underestimations of likeability (La Greca & Lopez, 1998; Van Zalk et al., 2011).

The second research question focused on how social anxiety symptoms in adolescents relate to self- and peer-reported friendships and the perception accuracy of friendships within the classroom. To answer this question, we first focused on the association of social anxiety with the number of self-nominated, peer-nominated, and reciprocal friends (Goal 2a). We computed discrepancies between these three scores as indicators of underestimation and overestimation of the number of friendships. Subsequently, we examined how these discrepancies were related to social anxiety symptoms (Goal 2b). We expected that higher levels of social anxiety would be related to fewer self-reported friendships (La Greca & Lopez, 1998; Van Zalk et al., 2011), fewer peer-reported friendships, and fewer reciprocal friendships. We also expected that adolescents would underestimate the number of friendships (i.e., them ‘being blind’ to the friendship offered by others). With regards to the subcategories of social anxiety, we again expected that especially the behavioral symptoms (avoidance) would be associated with the number of self-reported and peer-reported friends. In contrast, the cognitive symptoms would be associated with underestimating the number of friendships.

## Method

### Participants and Procedure

A total of 263 adolescents (49.4% boys) between 10 and 15 years old from 12 classrooms in Grades 7 and 8 from two secondary schools (*M* = 13.64, *SD* = 0.65) participated in the study. In total, 168 adolescents (63.9%) attended the school level ‘senior general secondary education / pre-university education,’ and 95 adolescents (36.1%) attended the school level ‘pre-vocational secondary education / senior general secondary education.’ The adolescents completed digital questionnaires in a classroom setting. Tables were set up in a ‘test’ setting so that participants could not see each other’s answers. Also, the project coordinator stressed that there were no right or wrong answers and that the individual results would not be shared with anyone. The study was part of a larger study on social development and bullying (Henricks et al., 2021; Pouwels et al., 2018, 2019). The Ethical Committee of the Behavioural Science Institute of Radboud University Nijmegen, the Netherlands, approved this study.

### Materials

#### Social Anxiety Symptoms

Social anxiety symptoms were measured with the shortened version of the *Social Anxiety Scale for Adolescents* (SAS-A; La Greca & Lopez, 1998; Kärnä et al., 2010). This questionnaire consists of nine items divided into two subscales, ‘Fear of Negative Evaluation’ (FNE) and ‘Social Avoidance and Distress’ (SAD; derived from the original SAD-general subscale of the SAS-A). Adolescents were asked to indicate to what extent the questions applied to them, ranging from 0 (never) to 4 (always). The shortened version of the SAS-A has a good internal consistency, α = .88 (Kärnä et al., 2010). In our sample, the SAS-A also had a good internal consistency, α = .88. The subscale FNE had an excellent internal consistency, α = .93, and the subscale SAD-general had a good internal consistency, α = .84. Both the total scale and the two subscales were included in the analyses.

#### Likeability

The adolescents were asked to answer on a Likert scale (1 = not at all, 7 = a lot), “How much do you think your classmates like you?”. This score is indicated as *like-self*. All adolescents were also given a list of their classmates and asked to answer on a Likert scale (1 = not liked at all, 7 = very much liked): “How much do you like classmate X?”. The average received score for each participating adolescent was computed and indicated as *like-peer*.

Discrepancy scores were used as a measure of perception bias. The discrepancy scores were computed by subtracting the *like-peer*-score from the *like-self*-score. This resulted in the *like-discr-*score. Positive values correspond with overestimating one’s own likeability, and negative scores with underestimating one’s likeability.

#### Friendship

First, adolescents were asked to name up to five best friends among their classmates. In a second question, adolescents were asked to name who were other good friends among their classmates. *Best-friend*-scores were derived from the first question and *total-friend*-scores were derived by computing the sum of the first and second question. *Given*-*friend-*scores were derived by computing the number of indicated best friend and good friend nominations by each participant. *Received*-*friend-*scores were derived from the number of received nominations for each participant (i.e., how often a participant was indicated as a friend) from their classmates. Furthermore, for both the *best-friend-* and *total-friend-*scores the number of *reciprocal* nominations was determined by examining how often the participant and a classmate nominated each other as friends. This resulted in six friendship scores: *best-friend-received, best-friend-given, best-friend-reciprocal, total-friend-received, total-friend-given, total-friend-reciprocal.*

Over- and underestimation scores were computed to obtain information on the extent to which adolescents’ self-indicated friendships were in line with the friendships indicated by their classmates. These two types of scores were used as indicators for perception accuracy. The first type of perception bias indicator on friendship was the *underestimate-*scores. *Underestimate*-scores were computed by subtracting the *reciprocal*-scores from the *received-friend*-scores (*N_received_* – *N_reciprocal_*). These scores indicate to what extent an adolescent recognizes the friendship-nominations that they receive. Higher *underestimate*-scores correspond with higher underestimation of the number of friends in the class. The second type of perception bias scores on friendship are the *overestimate*-scores. These were computed by subtracting the *reciprocal*-scores from the *given-friend*-scores (*N_given_* – *N_reciprocal_*). These scores indicate the extent to which peers indicate the same friendships as the participants themselves nominate. Higher *overestimate*-scores correspond with higher levels of overestimation of the number of friends in the class. The *overestimate-* and *underestimate-*scores were both computed for the number of best friends and the number of total friends. In total, this resulted in four friendship accuracy scores: overest-best-friend, underest-best-friend, overest-total-friend, and *underest-total-friend.*

### Data Analysis

Before answering the research question, a Pearson correlation between the two subscales of the SAS-A (FNE and SAD) was computed. In addition, we tested if the *like-discr-*score deviated significantly from zero with a one-sample *t*-test to test if the group of adolescents overestimated or underestimated their likeability by peers on average.

To answer the first research question – how social anxiety symptoms in adolescents relate to the perception accuracy of likeability by peers – partial correlations were computed between social anxiety symptoms (SAS-A) and *like-self*, *like-peer*, and *like-discr*, while controlling for gender and age. We controlled for age and gender in the analyses since previous studies found evidence for the relation between age, gender, social anxiety symptoms, and biased perceptions (Baartmans et al., 2019, 2020). The partial correlation between the social anxiety symptoms and *like-self* and *like-peer* provides information about how the self-estimates and the opinions of peers about adolescents’ likeability are related to social anxiety symptoms. The partial correlation between *like-discr* and social anxiety symptoms indicates if adolescents with higher levels of social anxiety are more likely to underestimate their likeability by peers.

To answer the second research question – how social anxiety symptoms in adolescents relate to the perception accuracy of their friendships within the classroom – we first tested how social anxiety symptoms were related to the self- and peer-nominations of friendships within the class. Therefore, partial correlations were computed between social anxiety symptoms and the *best-friend-received, best-friend-given, best-friend-reciprocal, total-friend-received, total-friend-given,* and *total-friend-reciprocal-scores* while controlling for gender and age. In the next step, partial correlations were computed between social anxiety symptoms and *overestimate-best-friend, underestimate-best-friend, overestimate-total-friend,* and *underestimate-total-friend* to test if social anxiety symptoms in adolescents were related to over- and/or underestimation of the number of friendships, again while controlling for age and gender.

In order to discriminate between sub-symptoms of social anxiety in their relation to perception accuracy of likeability and friendship – we conducted all analyses described above for the total social anxiety symptoms, as well as for the FNE- and SAD-scale separately.

## Results

### Descriptives

The Pearson correlation between the FNE and SAD subscales was .35 (*p* < .001). The *like-discr-*score had a mean of 0, indicating that adolescents had, on average, an accurate perception of their own likeability as rated by their peers. However, the standard deviation suggests that some adolescents underestimated or overestimated their likeability (see Table 1).

**Table 1:** Means (M) and Standard Deviations (SD) of the Social Anxiety Symptoms, Likeability Measures, and Friendship Measures

Variable	*M*	*SD*
SAS-total	1.46	0.67
SAS-SAD	1.78	0.85
SAS-FNE	1.07	0.75
Like-self	4.34	0.81
Like-peer	4.29	0.57
Like-discr	0.00	1.28
Best-friend-given	4.14	1.24
Total-friend-given	7.98	3.98
Best-friend-received	4.00	2.09
Total-friend-received	7.68	3.18
Best-friend-reciprocal	2.79	1.36
Total-friends-reciprocal	5.62	2.63
Overest-best-friend	1.35	1.36
Overest-total-friend	2.36	2.80
Underest-best-friend	1.21	1.31
Underest-total-friend	2.06	1.95

*Note.* SAS-total = total anxiety symptoms; SAS-SAD = social avoidance and distress symptoms; SAS-FNE = fear of negative evaluation symptoms; like-self = self-perceived likeability; like-peer = peer rated likeability; like-discr = difference between self- and peer-perceived likeability; best-friend-given = self-nominated number of best friends; total-friend-given = self-nominated number of best and good friends; best-friend-received = peer-indicated nominations of best friend; total-friend-received = peer-indicated nominations of total friend; best-friend-reciprocal = reciprocal number of best friends; total-friend-reciprocal = reciprocal number of best and good friends; overest-best-friend = overestimation of number of best friends; overest-total-friend = overestimation of number of best and good friends; underest-best-friend = underestimation of number of best friends; underest-total-friend = underestimation of number of best and good friends.

### Research Question 1: Social Anxiety and Likeability

As expected, higher levels of social anxiety were significantly related to lower self-estimates of likeability (see Table 2). In addition, higher levels of total anxiety were also significantly related to being less likeable according to peers and an underestimation of likeability by peers. Adolescents with higher levels of social anxiety thus were less liked by their peers and also indicated themselves that their peers liked them less (Goal 1A). However, adolescents with higher social anxiety scores were, on average, too pessimistic about their low likeability, as indicated by a tendency to underestimate their actual likeability level (Goal 1B). As expected, both higher levels of the subscales Fear of Negative Evaluation and Social Avoidance and Distress were significantly related to lower self-estimates of likeability. In accordance with our hypothesis, only the avoidance subscale was significantly related to lower peer ratings of likeability. This resulted in a significant negative relation between the negative evaluation scale and the discrepancy score but not between the avoidance subscale and the discrepancy score. These results suggest that a higher fear of negative evaluation was related to the underestimation of likeability by peers. In contrast, higher levels of social avoidance were related to both lower peer- and self-ratings of likeability, resulting in unbiased perceptions of one’s likeability.

**Table 2:** Partial Correlations Between Social Anxiety Symptoms (Total, FNE, and SAD), the Likeability Measures, the Friendship Measures, and the Perception Bias Measures on Friendship While Controlling for Gender and Age

Variable	Social anxiety symptoms		
	Total	FNE	SAD
Likeability			
Like-self	-.31***	-.22***	-.32***
Like-peer	-.14*	-.04	-.23*
Like-discr	-.13*	-.14*	-.07
Friendship quantity			
Best-friend-given	-.19*	-.14	-.18*
Total-friend-given	-.11**	-.07	-.13*
Best-friend-received	-.16*	-.04	-.25***
Total-friend-received	-.10	.02	-.22***
Best-friend-reciprocal	-.17*	-.06	-.25***
Total-friends-reciprocal	-.17*	-.06	-.24***
Friendship accuracy			
Overest-best-friend	.00	-.07	.08
Overest-total-friend	.00	-.04	.04
Underest-best-friend	-.08	.00	-.14*
Underest-total-friend	.07	.11**	-.02

*Note.* Total = total anxiety symptoms; FNE = fear of negative evaluation symptoms; SAD = social avoidance and distress symptoms; like-self = self-perceived likeability; like-peer = peer rated likeability; like-discr = difference between self- and peer-perceived likeability; best-friend-given = self-nominated number of best friends; total-friend-given = self-nominated number of best and good friends; best-friend-received = peer-indicated nominations of best friend; total-friend-received = peer-indicated nominations of total friend; best-friend-reciprocal = reciprocal number of best friends; total-friend-reciprocal = reciprocal number of best and good friends; overest-best-friend = overestimation of number of best friends; overest-total-friend = overestimation of number of best and good friends; underest-best-friend = underestimation of number of best friends; underest-total-friend = underestimation of number of best and good friends.

*p < .05. **p < .01. ***p < .001.

### Research Question 2: Social Anxiety and Friendship

As expected, total social anxiety symptoms were significantly related to self-nominating fewer best friends, being less often nominated as best friends, and fewer reciprocal best friends and reciprocal total friend nominations (Goal 2a). No significant relations existed between the Fear of Negative Evaluation subscale and friendship quantity measures. In contrast, the Social Avoidance and Distress subscale was significantly related to lower scores on all friendship quantity measures. Thus, higher levels of avoidance and distress were related to less self-nominated, peer-nominated, and mutual best friends and total friends. In contrast, fear of negative evaluation was not (see Table 2).

The results of the partial correlations between the over- and underestimation scores and social anxiety symptoms are displayed in Table 2 (Goal 2b). Total social anxiety symptoms and fear of negative evaluation were not significantly related to over- or underestimation of best friends and total friends. Only the subscale of social avoidance and distress was negatively related to the *underestimate-best-friend-*score, which suggests that adolescents with high levels of social avoidance and distress were less likely to “miss” best friends they had within their class than adolescents with low levels of social avoidance and distress. In other words, when adolescents show higher levels of avoidance and distress, they are more likely to recognize a friendship.

## Discussion

The overall aim of the current study was to investigate how different symptoms of social anxiety in adolescents relate to adolescents’ perception accuracy of likeability by peers and friendships with their peers. The first goal was to examine how social anxiety symptoms in adolescents are related to self-perceived likeability, peer-perceived likeability, and the perception accuracy in likeability. The second goal was to examine how social anxiety symptoms relate to self-perceived friendships, peer-perceived friendships, and perception accuracy of friendships within the classroom. As social anxiety can be divided into cognitive factors or symptoms (i.e., worry about negative evaluation) and behavioral symptoms (i.e., avoidance; American Psychiatric Association, 2013; Chavira & Stein, 2005; Clark & Wells, 1995; Ranta et al., 2014), we differentiated between cognitive and behavioral anxiety symptoms in the examination of both research goals.

### Social Anxiety and Likeability

We first examined how overall levels of social anxiety were related to self-rated and peer-rated likeability (Goal 1a). As hypothesized, adolescents with higher levels of social anxiety were less liked by their peers according to themselves and their classmates. This finding is in line with other studies that also found that higher social anxiety symptoms were related to lower peer-rated likeability (Baartmans et al., 2020; Verduin & Kendall, 2008) and with studies finding that more social anxiety symptoms were related to decreased peer acceptance and victimization in adolescents (Henricks et al., 2021; Tillfors et al., 2012; Verduin & Kendall, 2008). We also examined the discrepancy between adolescents’ own likeability perception and the likeability perception of their peers (Goal 1b). As expected, socially anxious adolescents tended to underestimate their likeability. These findings are in line with previous findings showing that social anxiety symptoms in pre-adolescents and adolescents with mild intellectual disability are related to the underestimation of likeability by peers (Baartmans et al., 2019; Klein et al., 2018). Therefore, by studying a sample of 10- to 15-year-old typically developing adolescents, the current study found that adolescents’ overall levels of anxiety were not only related to actual lower likeability according to peers but also to an underestimation of their likeability.

An important note is that most previous studies did not discriminate between worrying about negative judgment by others and the tendency to avoid social situations as subtypes of social anxiety when studying the accuracy of perceived likeability among peers. In order to overcome this limitation, we also examined how the cognitive and behavioral social anxiety symptoms were uniquely associated with self-perceived likeability, peer-perceived likeability, and the accuracy of perceived likeability. As expected, a higher *tendency to avoid* social situations was associated with being less liked by peers and perceiving oneself as less liked. Therefore, adolescents with a higher tendency to avoid social situations accurately perceive that their peers like them less than other classmates. In contrast, although adolescents with a *tendency to worry* about social situations (fear of negative evaluation) had the perception that they were less liked among their peers, their social anxiety symptoms were unrelated to their actual likeability among peers. Adolescents with cognitive and social anxiety symptoms, therefore, have an inaccurate perception (i.e., underestimation) of one’s likeability among peers.

Like Henricks and colleagues (2021, 2023), this study shows that especially avoiding social situations is associated with low likeability among peers. It is known that the innate need to belong becomes more important in adolescence and that adolescents might prefer peers with less strong avoidance tendencies (Baumeister & Leary, 1995; Schoch et al., 2015). At the same time, avoidance tendencies become stronger and more influential in adolescence (Miers et al., 2014), which could explain our finding that the tendency to avoid is related to less likeability by peers. In addition, worrying mainly plays a role in the mind, while others can notice avoidance tendencies, which may lead to lower ratings on likeability.

### Social Anxiety and Friendships

Regarding friendships (Goal 2), the results of the current study confirmed the hypothesis and previous research findings that social anxiety is related to fewer friendships (La Greca & Lopez, 1998; Van Zalk et al., 2011). We extended these findings by showing that like for likeability, these findings depend on the type of social anxiety symptoms. In line with our hypothesis, the tendency to avoid social situations and/or experiencing social distress was related to fewer (self-reported, peer-reported, and reciprocal) friendships. In contrast, worrying about negative judgment by others was unrelated to self-perceived and peer-perceived and reciprocal friendships.

In contrast to the findings for likeability and the hypothesis, total levels of social anxiety symptoms were *not* significantly related to more over- or underestimation of the number of best friends and total friends. Thus, adolescents with higher levels of social anxiety did not seem to have a biased perception of the number of friendships within the class compared to adolescents with lower levels of social anxiety. Surprisingly, a stronger tendency to avoid social situations and/or experiencing social distress was related to less underestimation of the number of best friends within the class, whereas worry was not associated with a biased friendship perception.

Based on theories about cognitive biases in social anxiety, we expected that socially anxious adolescents might underestimate their number of friendships (Hofmann & DiBartolo, 2014; Morrison & Heimberg, 2013), but the results did not show this. The lack of a significant relation between social anxiety symptoms and underestimating the number of one’s friendships might be explained by the fact that friendship is a social construct that requires reciprocal liking and, for instance, engaging in joint activities (Bukowski & Hoza, 1989; Demir & Urberg, 2004). These requirements could make it easier and clearer for adolescents to be sure whether or not someone can be considered as a friend. Thus, the fact that friendship quantity might be easier to perceive than liking could possibly explain why adolescents with higher levels of (cognitive) social anxiety seem to have more problems with accurately estimating a more general concept, such as their likeability, and not with a more concrete concept, such as friendship.

### Strengths, Limitations, and Suggestions for Future Research

A strength of the current study was the differentiation of the social anxiety symptoms into the subscales of fear of negative evaluation and the tendency to avoid social situations. This provides further information about the specific relation between social anxiety symptoms and social functioning measures. An additional strength was using a multiple-informant approach and including different measures of likeability and friendship. Limitations also need to be mentioned.

A first limitation of the current study was that we included a typically developing community sample only and no clinical sample. Even though the levels of social anxiety were comparable to other community sample studies (Kärnä et al., 2010), the scores of social anxiety were relatively low. This limits the generalization of the results to children with high levels of social anxiety or clinical samples. Clearly, more research is needed on children with high levels of social anxiety and clinical samples to draw more robust conclusions and to recommend implications for treatment. Second, the current study used a cross-sectional design. Therefore, no conclusions can be drawn about the causality or longitudinal effects of social anxiety symptoms and perception accuracy of social functioning. Third, using self-reports in a classroom setting could have led to socially desirable answers. Adolescents filled in the questionnaires in a ‘test setting’ so that others could not see their answers, and we stressed that there were no right or wrong answers and that the results would not be shared with anyone. Still, we cannot rule out the fact that social desirability might have influenced our findings. Fourth, there were some limitations regarding our friendship measures. We only measured friendships within the classrooms. Therefore, we cannot generalize these results to friendships in general. It could, for instance, be that socially anxious adolescents have more friendships outside the classroom (e.g., friends from primary school or sports).

The findings of the current study lead to some suggestions for future research. First, as we only included adolescents between the ages of 10-15 in the current study, little is known about how the associations between social anxiety, likeability, and friendships vary by age. A broader age range would allow drawing further conclusions regarding the relation between social anxiety symptoms and perceptions of social functioning across ages. Second, the current study extended previous research using a multi-method approach using self- and peer-reported measures. This approach could be further strengthened by including measures of social anxiety and social functioning by other raters, like teachers and parents. Third, a suggestion for future research would be to include measures to examine depression and externalizing problems. As depression and externalizing disorders are found to influence likeability and friendship and often co-occur with social anxiety disorder (Mohammadi et al., 2020), it would be especially important to include both variables when testing the associations between social anxiety, likeability, and friendships, to get a better understanding of the complex constructs of likeability and friendships in relation to mental health. Fourth, further research could be conducted to get more fine-grained insights into the accuracy of perceptions of adolescents’ friendships. In this study, we determined over- and underestimating friendships by studying discrepancies in nominations of friendships within the classroom. This differed from the likeability accuracy scores since we did not ask who the adolescent thought would indicate them as friends. This would have allowed us to distinguish adolescents not noticing others wanting to be friends with them from adolescents noticing a peer wanting to be friends with them but choosing not to engage in a friendship with that peer. Including these measures in future research would allow for drawing more precise conclusions on the accuracy of estimations.

### Practical Implications

The current study stresses the importance of targeting avoidance behavior in treating social anxiety symptoms. When adolescents with higher levels of social anxiety report the tendency to avoid, they are more likely to be disliked by peers, which could make their socially anxious thoughts about being negatively evaluated by others warranted. If replicated in a sample with high levels of social anxiety, these results might suggest that in addition to the cognitive training in CBT, encouraging socially anxious adolescents to approach and practice social situations rather than avoid them appears to be an important aspect of treatment. In addition, following the Extended Process Model (Gross, 2015), it may be interesting to examine which of the five ways (situation selection, situation modification, attentional deployment, cognitive change, and/or response modulation) is the most relevant (or best) target for treating social anxiety.

### Conclusion

In conclusion, the results of the current study provide evidence that even though social anxiety symptoms are related to both lower self-estimates and peer-estimates of likeability, adolescents with higher levels of social anxiety still seem to underestimate their likeability among classroom peers. In addition, we found that adolescents who worried more about negative evaluations underestimated their likeability by peers; they think they are less liked than they are. No such effects occurred for worrying about negative evaluations and perceptions of friendship. Further, adolescents with strong social avoidance tendencies accurately perceived their likeability and friendship; they were less liked by their peers and had fewer friendships, and they also perceived their situation as such. Even though these findings must be reviewed in light of the current study's limitations, these results highlight the possible importance of disentangling different subtypes of social anxiety and using multiple informants of likeability and friendships in future research and clinical practice. If replicated in a group of children with high (clinical) levels of social anxiety, these results emphasize the importance of explicitly treating avoidance behavior in social anxiety since this may not only maintain the social anxiety symptoms but were also found to be related to more negative judgments by others.

## Data Availability

Data from our study is available by contacting the corresponding author.

## Preregistration

There was no preregistration of the study.

## Reporting Guidelines

We followed APA7 reporting guidelines and JARS-Quant guidelines for writing the manuscript.

## Social Media Accounts

@ReinoutWiers, @SusanBogels

## Related Versions

The manuscript was based on Chapter 8 of the dissertation of the first author:

Baartmans, J. M. D. (2021). *Childhood social anxiety: What’s next? Exploring the role of cognitions, depression, parents, and peers*. University of Amsterdam. URL: https://pure.uva.nl/ws/files/54463245/Thesis_complete_.pdf
